# Primary dilated cardiomyopathy with LAMA2 and PKP4 mutations: imaging, genetics, and histology

**DOI:** 10.1093/ehjcr/ytag148

**Published:** 2026-03-05

**Authors:** Fang Zhou, Jinghui Li, Minjie Lu

**Affiliations:** Branch of Magnetic Resonance Imaging, Department of Radiology Center of Imaging, Fuwai Hospital and National Center for Cardiovascular Diseases, Chinese Academy of Medical Sciences and Peking Union Medical College, Beilishi Road No. 167, Xicheng District, Beijing 100037, China; Department of Radiology, The People’s Hospital of Xishuangbanna Dai Nationality Autonomous Prefecture, No. 4, Galan South Road, Jinghong City, Xishuangbanna Dai Autonomous Prefecture, Yunnan Province 666100, China; Branch of Magnetic Resonance Imaging, Department of Radiology Center of Imaging, Fuwai Hospital and National Center for Cardiovascular Diseases, Chinese Academy of Medical Sciences and Peking Union Medical College, Beilishi Road No. 167, Xicheng District, Beijing 100037, China; Branch of Magnetic Resonance Imaging, Department of Radiology Center of Imaging, Fuwai Hospital and National Center for Cardiovascular Diseases, Chinese Academy of Medical Sciences and Peking Union Medical College, Beilishi Road No. 167, Xicheng District, Beijing 100037, China

A 25-year-old woman presented with a 1-year history of intermittent fever and sore throat, followed by progressive chest tightness and palpitations. Electrocardiography demonstrated first-degree atrioventricular block, abnormal atrial depolarization suggestive of atrial electrical remodelling, ST segment and T wave changes (*Panel A*), and episodes of atrial fibrillation and atrial flutter during hospitalization. Holter monitoring demonstrated a low burden of ventricular ectopy (13 premature ventricular contractions per day) and frequent supraventricular ectopy (158 premature supraventricular beats per day), without sustained ventricular tachycardia. During hospitalization, atrial fibrillation, atrial flutter, and intraventricular conduction delay were observed. Laboratory testing revealed markedly elevated N-terminal prohormone of brain natriuretic peptide levels (27 718 pg/mL). Coronary computed tomographic angiography (CTA) excluded significant coronary artery stenosis (*Panel B*). Family history was negative for cardiomyopathy, heart failure, arrhythmia, or sudden cardiac death. The patient was classified as New York Heart Association (NYHA) functional Class IV. Despite guideline-directed medical therapy for heart failure over 1 year, she experienced recurrent episodes of acute decompensated heart failure. During the current admission, progressive haemodynamic deterioration culminated in cardiogenic shock, requiring intra-aortic balloon pump support. These findings, together with severe biventricular systolic dysfunction, pulmonary hypertension, and persistently elevated natriuretic peptide levels, were consistent with advanced heart failure.

**Figure:**
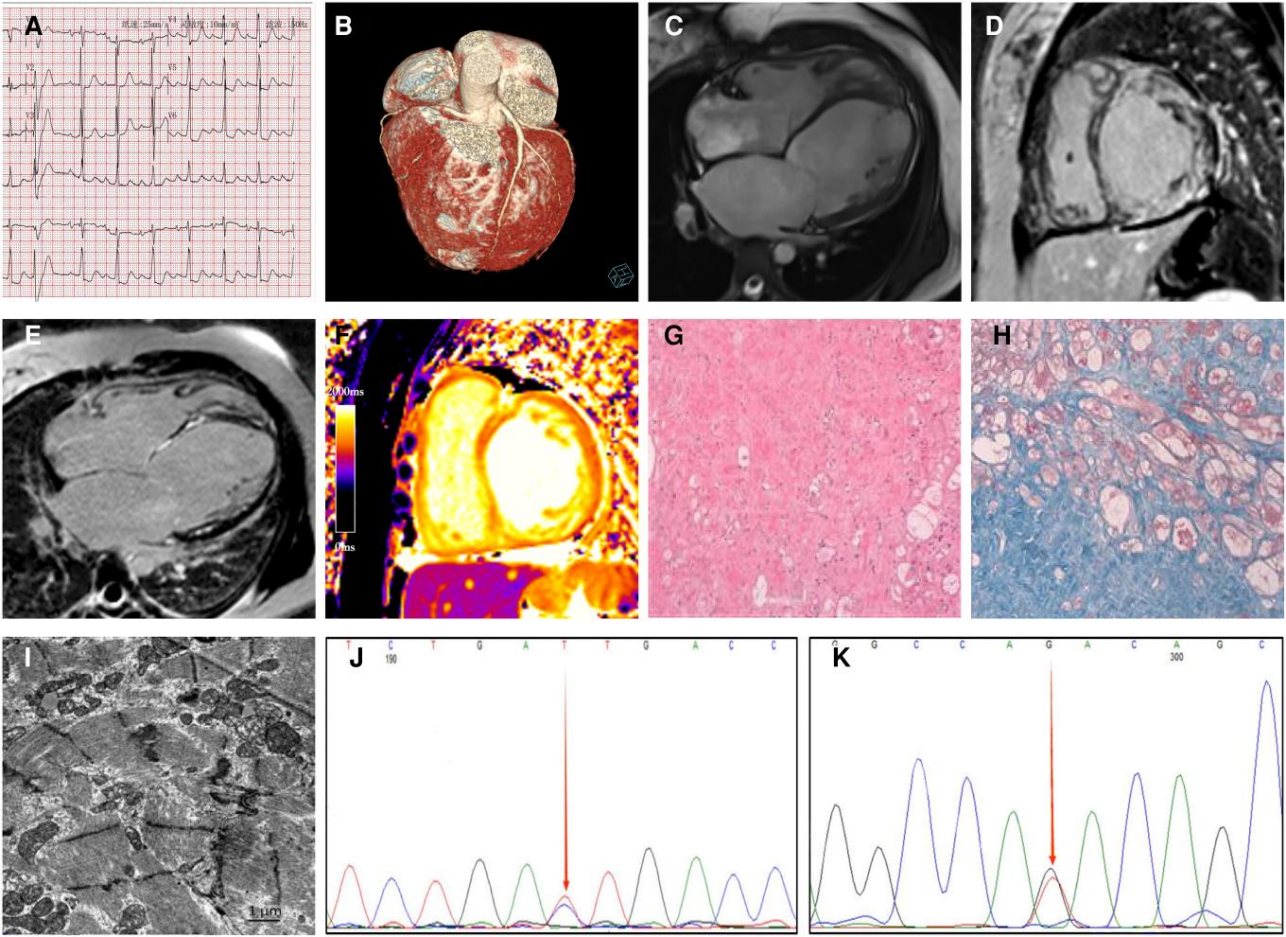


Transthoracic echocardiography at admission demonstrated global cardiac enlargement with diffuse left ventricular hypokinesia and markedly reduced systolic function (left ventricular ejection fraction 33%). The interventricular septum and left ventricular wall were diffusely thinned, with reduced wall thickening throughout the cardiac cycle. Left ventricular end-diastolic diameter measured 61 mm. No focal regional wall motion abnormalities were identified. Reduced systolic excursion of the mid-to-apical right ventricular free wall was also noted. In addition, moderate mitral regurgitation, mild-to-moderate tricuspid regurgitation, and pulmonary hypertension (mean pulmonary artery pressure > 36 mmHg) were present.

Cardiac magnetic resonance (CMR) demonstrated four-chamber enlargement with biventricular systolic dysfunction (*Panel C*). Late gadolinium enhancement (LGE) revealed multiple subendocardial linear enhancements in the interventricular septum and lateral wall, as well as transmural fibrosis in the mid-anterior and inferior segments of the left ventricle (*Panels D–E*). Native T1 mapping showed diffusely elevated values (1416–1496 ms) (*Panel F*), indicating widespread myocardial fibrosis. Pulmonary hypertension, moderate-to-severe mitral regurgitation, and moderate tricuspid regurgitation were also present.

Due to advanced heart failure, the patient underwent orthotopic heart transplantation. Histopathology revealed non-specific ultrastructural changes consistent with primary dilated cardiomyopathy (DCM), including marked hypertrophy, vacuolar degeneration, and focal disarray. Electron microscopy showed extensive myofibrillar dissolution (*Panels G–I*). Whole-exome sequencing revealed heterozygous variants in the LAMA2 gene (c.1637C > T) and the PKP4 gene (c.1141G > T), both of which were classified as variants of uncertain significance (VUS; American College of Medical Genetics and Genomics (ACMG) Class II) based on the ACMG criteria, supported by the PM2 (Supporting) evidence (*Panels J–K*).

## Discussion

From a clinical perspective, this case illustrates how diagnostic reasoning evolved in parallel with the patient’s clinical course and multimodality testing. The initial presentation with chest symptoms, arrhythmias, and mildly elevated cardiac biomarkers raised suspicion for myocarditis. However, the persistence and progression of symptoms despite guideline-directed medical therapy, together with recurrent arrhythmias and worsening haemodynamics, prompted reconsideration of the underlying diagnosis. Transthoracic echocardiography provided the initial evidence of severe ventricular dysfunction, functional mitral and tricuspid regurgitation, and pulmonary hypertension, while CMR subsequently clarified the underlying myocardial aetiology and extent of fibrosis, highlighting the complementary role of multimodality imaging.

Cardiac magnetic resonance played a pivotal role in this diagnostic transition. Rather than demonstrating focal or transient inflammatory changes typical of acute myocarditis, CMR revealed diffuse myocardial fibrosis involving multiple left ventricular segments, markedly elevated native T1 values, and biventricular systolic dysfunction. These findings were more consistent with a chronic and progressive non-ischaemic cardiomyopathy than with a self-limited inflammatory process.

Late gadolinium enhancement provided crucial aetiological information. The LGE pattern was predominantly subepicardial and patchy, extending beyond a single coronary artery territory, which is a characteristic of non-ischaemic myocardial injury and makes an ischaemic cause highly unlikely, particularly in the absence of significant coronary artery disease on coronary CTA. In contrast to the subendocardial or transmural LGE following a coronary distribution seen in ischaemic cardiomyopathy, subepicardial and mid-wall enhancement is typically observed in inflammatory cardiomyopathy and dilated cardiomyopathy (*Table 1*). Thus, the LGE pattern supported a non-ischaemic aetiology and contributed to the differential diagnosis between myocarditis-like injury and progressive dilated cardiomyopathy. No definite fibro-fatty replacement was identified on CMR or histology in this patient. This case highlights that the absence of fibro-fatty replacement does not preclude desmosomal-related cardiomyopathy and underscores the phenotypic heterogeneity of these conditions, particularly in young patients presenting with inflammatory activity and left ventricular involvement.

**Table 1:** Differential diagnosis of cardiomyopathies based on LGE distribution

Disease entity	Typical LGE distribution	Coronary territory	Teaching point
Ischaemic cardiomyopathy	Subendocardial or transmural	Yes	Follows coronary anatomy
Acute myocarditis	Subepicardial, often inferolateral	No	Inflammatory pattern
Dilated cardiomyopathy	Mid-wall or patchy	No	Fibrosis related to remodelling
Arrhythmogenic cardiomyopathy	Subepicardial or ring-like	No	Often involves LV inferolateral wall
Cardiac amyloidosis	Diffuse subendocardial	No	Difficulty nulling myocardium
Sarcoidosis	Patchy, multifocal	No	Non-coronary, variable

Assessment of myocardial oedema using T2-weighted short tau inversion recovery (STIR) imaging and T2 mapping further suggested intermittent inflammatory activity. Although T2 mapping values were only mildly elevated (38–46 ms), likely influenced by the markedly thinned ventricular wall and partial volume effects, increased signal intensity on STIR imaging was consistent with myocardial oedema. Importantly, a prior CMR examination performed in March 2024 had been interpreted as myocarditis, after which the patient experienced a marked decline in functional capacity. This temporal association supports the concept of intermittent ‘hot phases’ during cardiomyopathy evolution, which may accelerate myocardial remodelling and contribute to disease progression and worsening heart failure.

As the patient developed recurrent episodes of acute decompensated heart failure culminating in cardiogenic shock requiring intra-aortic balloon pump support, the clinical focus shifted from diagnostic clarification to advanced heart failure management. The integration of longitudinal clinical evolution, echocardiographic findings, CMR tissue characterization, laboratory abnormalities, and genetic data exemplifies a stepwise diagnostic reasoning process and underscores the central role of CMR in guiding clinical decision-making in complex cardiomyopathy cases.

Although variants in PKP4 and LAMA2 were identified, both were classified as VUS according to ACMG criteria. Variants in LAMA2 have been associated with a cardiomyopathic phenotype characterized by conduction abnormalities, fragmented QRS complexes, pathological Q waves, delayed R-wave progression, and impaired myocardial mechanics, including reduced global longitudinal strain and reduced left ventricular ejection fraction.

In the present case, the patient exhibited advanced left ventricular systolic dysfunction with markedly reduced left Ventricular Ejection Fraction and conduction abnormalities; however, classical electrocardiographic features, such as frequent pathological Q waves or overt QRS fragmentation, were not prominent. Given that the identified LAMA2 variant was classified as a VUS, its role is best interpreted as a potential phenotypic modifier rather than a primary causal factor.

Overall, this case underscores the central role of CMR in integrating structural, functional, and tissue-level information to guide diagnosis and management in complex cardiomyopathy.

## Data Availability

The data underlying this article are not publicly available due to patient privacy considerations.

## Notes

**Consent:** The authors confirm that written consent for submission and publication of this case report, including images and associated text, has been obtained from the patient in line with the COPE guidelines.

